# Topography and Higher Order Corneal Aberrations of the Fellow Eye in Unilateral Keratoconus

**DOI:** 10.4274/tjo.45220

**Published:** 2017-10-27

**Authors:** Sibel Aksoy, Sezen Akkaya, Yelda Özkurt, Sevda Kurna, Banu Açıkalın, Tomris Şengör

**Affiliations:** 1 Fatih Sultan Mehmet Training and Research Hospital, Ophthalmology Clinic, İstanbul, Turkey; 2 Bilim University Faculty of Medicine, Department of Ophthalmology, İstanbul, Turkey

**Keywords:** Corneal aberrations, topography, unilateral keratoconus

## Abstract

**Objectives:**

Comparison of topography and corneal higher order aberrations (HOA) data of fellow normal eyes of unilateral keratoconus patients with keratoconus eyes and control group.

**Materials and Methods:**

The records of 196 patients with keratoconus were reviewed. Twenty patients were identified as unilateral keratoconus. The best corrected visual acuity (BCVA), topography and aberration data of the unilateral keratoconus patients’ normal eyes were compared with their contralateral keratoconus eyes and with control group eyes. For statistical analysis, flat and steep keratometry values, average corneal power, cylindrical power, surface regularity index (SRI), surface asymmetry index (SAI), inferior-superior ratio (I-S), keratoconus prediction index, and elevation-depression power (EDP) and diameter (EDD) topography indices were selected.

**Results:**

Mean age of the unilateral keratoconus patients was 26.05±4.73 years and that of the control group was 23.6±8.53 years (p>0.05). There was no statistical difference in BCVA between normal and control eyes (p=0.108), whereas BCVA values were significantly lower in eyes with keratoconus (p=0.001). Comparison of quantitative topographic indices between the groups showed that all indices except the I-S ratio were significantly higher in the normal group than in the control group (p<0.05). The most obvious differences were in the SRI, SAI, EDP, and EDD values. All topographic indices were higher in the keratoconus eyes compared to the normal fellow eyes. There was no difference between normal eyes and the control group in terms of spherical aberration, while coma, trefoil, irregular astigmatism, and total HOA values were higher in the normal eyes of unilateral keratoconus patients (p<0.05). All HOA values were higher in keratoconus eyes than in the control group.

**Conclusion:**

According to our study, SRI, SAI, EDP, EDD values, and HOA other than spherical aberration were higher in the clinically and topographically normal fellow eyes of unilateral keratoconus patients when compared to a control group. This finding may be due to the mild asymmetric and morphologic changes in the subclinical stage of keratoconus leading to deterioration in the indicators of corneal irregularity and elevation changes. Therefore, these eyes may be exhibiting the early form of the disease.

## INTRODUCTION

Keratoconus is a noninflammatory corneal disease that generally exhibits bilateral and asymmetrical involvement. It results in thinning of the corneal stroma, corneal ectasia, irregular astigmatism, and reduced vision.^1,2^ The progressive course of the disease ultimately affects both eyes, though only one eye may be affected initially. The prevalence of true unilateral keratoconus has been reported to range from 0.5-4% in studies using computerized videokeratography^3,4,5,6^ and was 4.5% in a more recent study using slit scanning corneal topography (Orbscan 2).^7^ Holland et al.^5^ reported that patients with unilateral keratoconus developed signs of keratoconus in their apparently healthy fellow eyes 4 years later, while Li et al.^8^ found that keratoconus developed in 50% of cases within 16 years. Therefore, it may be concluded that the fellow eyes of patients with unilateral keratoconus may seem normal with regard to clinical and topographical patterns yet have subclinical keratoconus. While it is easy to diagnose moderate and advanced keratoconus based on typical clinical and topographical findings, the lack of definitive criteria makes it difficult to diagnose subclinical keratoconus in patients who have normal visual acuity and do not exhibit clinical findings. This is particularly important in examinations prior to refractive surgery, as ectatic corneal disorders that have not been identified before refractive surgery may result in progressive keratectasia. Placido disk-based corneal topography is one of the methods commonly used to diagnose keratoconus. Many numerical topographic indices which identify abnormalities in keratoconic cornea topography have been developed, and these indices have high sensitivity and specificity in diagnosing keratoconus.^8,9,10,11^

The anterior surface of the cornea is the most important refractive component of the eye, and high-order corneal aberrations are seen significantly more in keratoconic corneas than in normal corneas. It has been reported that considering corneal topography in combination with corneal wavefront aberrations when diagnosing keratoconus may result in higher detection rates.^12,13^

The aim of this study was to analyze the quantitative topography indices and corneal high-order aberration (HOA) data from the normal eyes of unilateral keratoconus patients and to compare them with the fellow keratoconic eye and with the normal eyes of healthy individuals.

## MATERIALS AND METHODS

The records of 392 eyes of 196 patients diagnosed with keratoconus in the cornea and contact lens unit of our clinic between 2008 and 2015 were retrospectively reviewed. Twenty patients with clinical and topographical keratoconus in one eye and no clinical or topographical keratoconus findings in the fellow eye were included. The eyes were divided into the unilateral keratoconus group and a normal fellow eye group. Each of the patients was diagnosed with keratoconus based on a collective assessment of refraction examination, slit-lamp anterior segment examination, and corneal topography. The normal eyes of these patients had a keratometric astigmatism below 1.5 diopter (D), vertical keratometry value below 47 D, and no keratoconus patterns such as asymmetrical bow-tie pattern, skewed axis, or localized steepening on topography. The control group consisted of the right eyes of 20 age-matched healthy individuals without any ocular pathology except for refractive errors. Patients with ocular surgery history or accompanying ocular pathology were excluded from the study.

For all patients, best corrected visual acuity (BCVA) on Snellen chart, corneal topography and corneal HOA data were recorded. Corneal topography and cornea aberration measurement results were obtained from the database of the Placido-based NIDEK Magellan Mapper (NIDEK Technologies Srl, Padova-Italy) topography system. Flat (K1) and steep (K2) keratometry values, average corneal power (ACP), cylindrical power (CYL), surface regularity index (SRI), surface asymmetry index (SAI), inferior/superior ratio (I-S), keratoconus prediction index (KPI), elevation-depression power (EDP), and elevation/depression diameter (EDD) topography indices were selected for statistical analysis. BCVA, quantitative topography indices, and HOA root mean square (RMS) values of the normal fellow eyes were compared with those of the keratoconic eyes and the control eyes.

### Statistical Analysis

Statistical analysis was carried out using NCSS (Number Cruncher Statistical System) 2007 & PASS (Power Analysis and Sample Size) 2008 Statistical Software (Utah, USA) software. The study data were evaluated using descriptive statistical methods (mean, standard deviation, median) as well as independent-samples test for intergroup comparison of parameters with normal distribution and the Mann-Whitney U test for intergroup comparisons of parameters with abnormal distribution. The results were assessed within a 95% confidence interval and significance was accepted at p<0.05.

## RESULTS

The prevalence of unilateral keratoconus among our patient group was 11.2%. The mean age of the 20 unilateral keratoconus patients was 26.05±4.73 years, and that of the 20 individuals in the control group was 23.60±8.53 years (p>0.05). 

BCVA was 0.47 in the keratoconus eyes, 0.97 in the normal fellow eyes, and 1.0 in the control eyes. Although K1, K2, ACP, and CYL values were only slightly higher in the normal fellow eyes when compared to the control eyes, the difference was statistically significantly (p<0.01). The keratoconic eyes had significantly higher K1, K2, ACP, and CYL values compared to the normal fellow eyes (p<0.01).

Comparison of topography indices revealed no difference in I-S between the normal and control groups (p=0.314), whereas SRI, SAI, KPI, EDP, and EDD values were significantly higher in the normal group compared to the control group (p<0.01). All topographic indices of the keratoconic eyes were significantly higher than those of the normal fellow eyes (p<0.01). BCVA, K1, K2, ACP, CYL, I-S, SRI, SAI, KPI, EDP, and EDD values of the groups are shown in Table 1, and the statistical significance levels are given in Table 2.

Evaluation of corneal aberrations showed no difference between the spherical aberration RMS values of the normal group and the control group (p=0.429), whereas coma, trefoil, irregular astigmatism, and total HOA-RMS values were significantly higher in the normal fellow eyes (p<0.01). However, all corneal aberrations in the keratoconic eyes were significantly greater than those of the normal fellow eyes (p<0.05). The HOA values of all patients are given in Table 3, with statistical significance levels in Table 4.

## DISCUSSION

Corneal topography is the gold standard in keratoconus diagnosis. There are various quantitative topography indices developed from computer-assisted videokeratoscopes which detect the topographic pattern of keratoconus. Some of these are the KPI, I-S index, KISA % index, and SARX (skewed radial axis) index, and these indices are highly sensitive in diagnosing keratoconus.^11,14^ Each is generally associated with a topography instrument. Software of the NIDEK Magellan Mapper corneal topography equipment used in our study includes indices that provide information about surface asymmetry and elevation changes, as well as I-S index and KPI. As in many other studies in the literature, our study also demonstrates that keratoconus eyes have significantly higher mean values in all indices when compared with normal fellow eyes and the control group.^11,13^

The I-S index, developed by Rabinowitz and McDonnell^14^, determines the dioptric power difference between the inferior and superior corneal zones. In the normal cornea, it is below 1.4. Patients with values above 1.4 are classified as suspected keratoconus, while values above 1.9, if accompanied by other clinical symptoms, are classified as keratoconus. Maximum I-S values are reached in decentralized cones, increasing relative to the displacement of the apex from the visual axis, and I-S values approach normal in centralized cones.^14^ Although in the present study the average I-S value of the normal fellow eyes was twice that of the control group, it was below 1.4. In a study by Tummanapalli et al.,^15^ the I-S value was 0.52 in the eyes with subclinical keratoconus, which was 2.6 times higher than in normal eyes. The authors concluded that I-S had low specificity and sensitivity in differentiating between keratoconic and normal corneas. We believe that this was because the dioptric power differences between inferior and superior zones have not yet been determined in subclinical keratoconus. In contrast, Gordon-Shaag et al.^13^ reported that the I-S ratio in subclinical keratoconus cases was 9.4 times higher than the normal cases, while Rabinowitz et al.^16^ found it to be 5.4 times higher. The former study included 21 subclinical keratoconus patients having astigmatism with symmetrical bow-tie pattern and a KCI over 35% without any other keratoconus symptoms, while the latter study included 16 subclinical keratoconus patients with a K value below 47 D and without inferior steepening. These cases exhibited more pronounced asymmetric characteristics compared to the cases in our study.

SRI indicates local fluctuations in central corneal power and is correlated with potential visual acuity. If SRI values are high, it may be attributable to corneal surface irregularities along the entrance pupil. Normal values are in the 0-0.56 range.^9,14,17^ Although in our study the mean SRI value in the normal fellow eyes was 0.55, it was significantly higher than that of the control eyes. SAI measures differences in corneal power within each ring along the entire corneal surface, and it increases as irregular astigmatism increases and the decentralized cone becomes steeper. Normal values are in the range of 0.10-0.42.^9,14,17^ In our study, the mean SAI value was 0.52 in the normal group and 0.33 in the control group. No research was found in the literature which used Placido-based topography to compare SRI and SAI in subclinical keratoconus and normal eyes. Lim et al.^11^ compared topographic indices in keratoconus and subclinical keratoconus cases using Placido-based topography (Tomey TMS-2) and reported a mean SRI of 0.7 and mean SAI of 1.04 in patients with subclinical keratoconus. In the same study, comparison of subclinical keratoconus cases and normal cases using slit-scanning topography (Orbscan 2) showed that 3-mm and 5-mm SRI values were significantly higher in the subclinical keratoconus group. In that study, the subclinical keratoconus group included patients who exhibited central, inferior, or superior steepening on topography in addition to over 1.5 D of oblique astigmatism, central corneal thickness less than 500 µm, and a steep keratometry value of 47 D without any biomicroscopic signs, whereas our group of normal fellow eyes had an average keratometry value of 44.23 D with no topographic signs that might indicate keratoconus. Similarly, Tummanapalli et al.^15^ used the Orbscan 2 to show that the anterior and posterior 3-mm and 5-mm SRI values were significantly higher in the subclinical keratoconus group than in the normal cases. Based on the findings of these studies, we can state that SRI and SAI are important indicators in the diagnosis of subclinical keratoconus.

The KPI was developed via multivariate analysis encompassing the SimK1, SimK2, OSI, CSI, DSI, SAI, IAI, and AA indices in order to improve diagnostic potential. As a composite index, it may be considered the most sensitive indicator for identifying cornea asymmetry. KPI values are below 0.225 in normal corneas.^14^ Smolek and Klyce^10^ reported that KPI values are not sufficient for distinguishing between moderate and severe keratoconus, and have limited value in determining the degree of asymmetric disease. In our study, the mean KPI value was within normal limits in the normal fellow eyes, but was significantly higher than that of the control group. Lim et al.^11^ reported a mean KPI of 0.27 in subclinical keratoconus, but the patients included in their study had topographic findings suggesting early stage keratoconus. In our patient group, the KPI values, like the I-S value, did not have any asymmetric features that may lead to the threshold value being exceeded. In that sense, the I-S and KPI indices are not sufficient by themselves to distinguish between subclinical keratoconus and normal eyes.

EDP calculates the average power of apparent islands and valleys for those areas of the cornea that are within the demarcated pupil. The unit is D. It can be used to estimate the size of so-called central islands after excimer laser photorefractive keratectomies. EDD is twice the square root of this zone divided by pi, is an equivalent diameter. The unit is mm. Abnormal EPD and EDD values can be seen in keratoconus, corneal grafts, and astigmatic normal corneas.^9^ In our study, the normal fellow eyes exhibited significantly higher EDP and EDD values compared to the control group. Our topography device provides only anterior elevation data and derives them from curvature maps. However, keratoconus affects not only the anterior surface of the cornea, but also the posterior surface. Uçakhan et al.^18^ reported that posterior elevation data were more definitive than anterior elevation data in distinguishing subclinical keratoconus from normal corneas. A study using Pentacam showed that 88% of the normal eyes of patients classified as unilateral keratoconus according to standard Rabinowitz criteria exhibited posterior corneal surface changes.^19^ It has also been determined that the posterior corneal surface shows changes before the anterior surface in ectatic corneal diseases.^20^ Rao et al.^21^ developed a keratoconus identification algorithm using posterior elevation values (Orbscan 2, cut-off 40 µm) in combination with videokeratography data (Rabinowitz or Klyce/Maeda method) and suggested that elevation and pachymetry data be combined with curvature data in cases of suspected keratoconus. Elevation-based topography systems provide both anterior and posterior cornea curvature and elevation maps, corneal thickness, and anterior segment data. Because the topography device used in the present study provides data about only the anterior surface of the cornea, it may not have been adequate to diagnose keratoconus.

Various studies have demonstrated that keratoconic corneas exhibit increased wavefront aberrations as compared to normal corneas.^22,23,24,25^ Zernike polynomials enable the rendering of complex corneal shapes and human eye wavefront aberrations to identifiable shapes and mathematical formulas such as defocus, astigmatism, and spherical aberration. RMS value shows the overall size of the aberration relative to pupil diameter; an optically perfect eye has an RMS value of 0. Our finding is consistent with the literature in that the RMS values of all HOAs in keratoconic patients were significantly higher than those of the normal fellow eyes and the control group. In the group of normal fellow eyes, all HOA-RMS values except spherical aberration were found to be higher than in the control group. Bühren et al.^26^ found that the coma, trefoil, total HOA-RMS values, and Z_3_^-1^, Z_5_^-1^ vertical coma coefficients were higher in the subclinical keratoconus group than in the normal cases, with the biggest difference being in the vertical coma coefficients. Accordingly, vertical asymmetry was interpreted to be the earliest symptom of keratoconus. Coma aberration results from the decentration of the optic system, clinically known as the kappa angle, and this natural condition manifests itself as a decentralized spherical aberration. In eyes with keratoconus, the cone with higher dioptric power than the other corneal surfaces causes deformation in wavefront, shifting of the visual axis and associated significant increase in coma aberration. Alio and Shabayek.^22^ determined that coma aberration was a good indicator in identifying and grading keratoconus; they observed a significant positive correlation between increasing K values and coma aberration, and developed a modified Amsler-Krumeich keratoconus grading system using coma aberration. Our study supported the results of Gordon-Shaag et al.,^13^ who showed that all HOAs other than total tetrafoil and spherical aberration were significantly higher in eyes with suspected keratoconus when compared with normal fellow eyes.

In our study, the unilateral keratoconus ratio was found to be 11.2%. The prevalence of true unilateral keratoconus is reported in the international literature as ranging between 0.5% and 4.5%. In a study conducted in Turkey, a unilateral keratoconus prevalence of 14.9% was determined using Pentacam.^27^ The main limitation of our study was that the elevation data provided by our topography device was not adequate and not able to evaluate the posterior corneal surface. Another limitation is that long-term patient follow-up data was not available. The suspected keratoconus eyes that we determined to be normal may exhibit signs that would lead to a keratoconus diagnosis if examined using more advanced topography systems.

## CONCLUSION

In summary, the present study demonstrates that in keratoconus cases, sometimes one of the eyes does not exhibit the clinical and topographical findings to diagnose keratoconus, but may be in a subclinical and subtopographical phase and have significantly different values compared to the eyes of normal people, particularly with regard to surface regularity indices, elevation values, and corneal HOAs. Therefore, we believe that the corneal curvature map, elevation map, and corneal HOAs should be evaluated collectively when diagnosing subclinical keratoconus and selecting eligible patients prior to refractive surgery.

## Figures and Tables

**Table 1:**
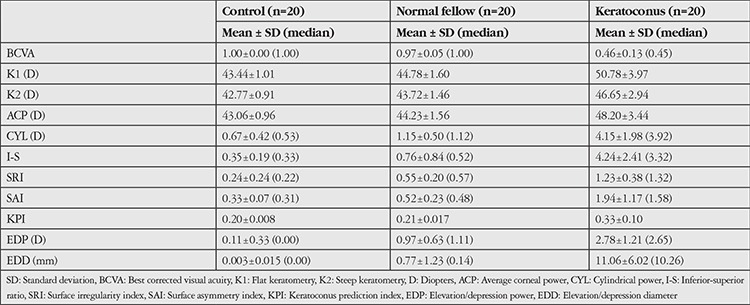
Best corrected visual acuity, K1, K2, average corneal power, cylindrical power, inferior-superior ratio, surface irregularity, surface asymmetry index, keratoconus prediction index, elevation/depression power, and elevation/depression diameter values in the study groups

**Table 2:**
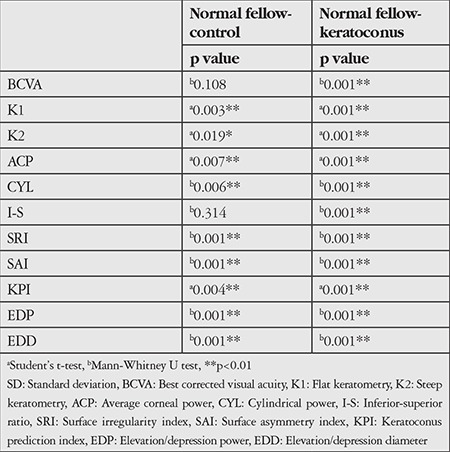
Comparison of best corrected visual acuity, K1, K2, average corneal power, cylindrical power, inferior-superior ratio, surface irregularity, surface asymmetry index, keratoconus prediction index, elevation/depression power, and elevation/depression diameter values of the study groups

**Table 3:**
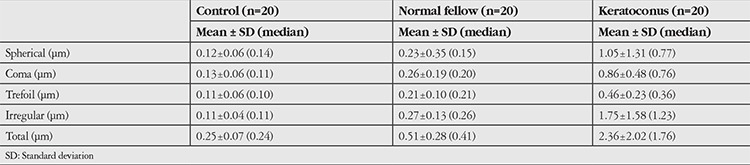
Distribution of high order aberrations by study group

**Table 4:**
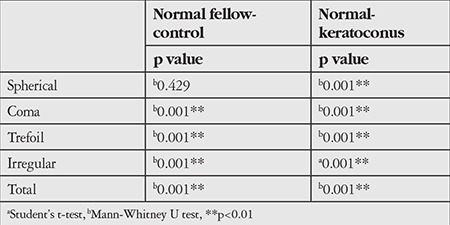
Comparison of high order aberrations of the study groups
